# Alcohol Consumption, One-Carbon Metabolites, Liver Cancer and Liver Disease Mortality

**DOI:** 10.1371/journal.pone.0078156

**Published:** 2013-10-28

**Authors:** Lauren M. Schwartz, E. Christina Persson, Stephanie J. Weinstein, Barry I. Graubard, Neal D. Freedman, Satu Männistö, Demetrius Albanes, Katherine A. McGlynn

**Affiliations:** 1 Division of Cancer Epidemiology and Genetics, National Cancer Institute, Rockville, Maryland, United States of America; 2 Department of Epidemiology Research, Statens Serum Institut, Copenhagen, Denmark; 3 Department of Chronic Disease Prevention, National Institute for Health and Welfare, Helsinki, Finland; University of Modena & Reggio Emilia, Italy

## Abstract

**Background:**

Excess alcohol consumption adversely affects one-carbon metabolism and increases the risk of liver disease and liver cancer. Conversely, higher folate levels have been inversely associated with liver damage. The current study investigated the effects of alcohol and one-carbon metabolite intake on liver cancer incidence and liver disease mortality within the Alpha-Tocopherol, Beta-Carotene Cancer Prevention (ATBC) Study.

**Methods:**

Cox proportional hazards modeling was used to calculate hazard ratios and 95% confidence intervals (CIs) in a population of 27,086 Finnish males with 194 incident liver cancers and 213 liver disease deaths. In a nested case-control subset (95 liver cancers, 103 controls), logistic regression was used to calculate odds ratios and 95% CIs for serum one-carbon metabolites in relation to liver cancer risk.

**Results:**

Daily alcohol consumption of more than 20.44 g was associated with an increased risk of both liver cancer incidence (Hazard Ratio (HR) 1.52, 95%CI 1.06–2.18) and liver disease mortality (HR 6.68, 95%CI 4.16–10.71). These risks were unaffected by one-carbon metabolite intake. Similarly, in the case-control study, none of the serum one-carbon metabolites were associated with liver cancer.

**Conclusions:**

The current study provided no convincing evidence for a protective association of one-carbon metabolite intake or serum level on the risk of liver cancer or liver disease mortality.

## Introduction

Excessive consumption of alcohol is a well-established risk factor for both liver disease and liver cancer [1]–[5]. Alcohol adversely affects the absorption of one-carbon metabolites, which can affect DNA methylation and lead to genomic instability and modified oncogene expression [6]–[8]. Folate and other one-carbon metabolites act as essential co-factors in enzymes that catalyze one-carbon transfer reactions [9]. For several cancers, folate and alcohol consumption have been jointly studied with varying results [10]–[12]. The risk of liver cancer and liver disease associated with one-carbon metabolites and alcohol, however, has not been widely investigated. Recently, a study by our group suggested that the effect of alcohol on increased risk of liver cancer incidence may be ameliorated by folate consumption [13].

The present study sought to assess the effect of alcohol consumption and one-carbon metabolite (folate, cysteine, vitamin B6, riboflavin, vitamin B12, and methionine) intake on liver cancer incidence and liver disease mortality in the Alpha-Tocopherol, Beta-Carotene Cancer Prevention (ATBC) Study. In addition, a nested case-control study examined the effects of serum levels of one-carbon metabolite levels and alcohol consumption on the development of liver cancer.

## Materials and Methods

### Ethics Statement

Written informed consent was provided by all study participants. The study was approved by the institutional reviews boards of the National Cancer Institute, Bethesda, Maryland, U.S., and the National Public Health Institute, Helsinki, Finland.

### Study Population

As previously described, the ATBC Cancer Prevention Study was conducted in Finland as a joint project between the U.S. National Cancer Institute and the National Public Health Institute of Finland [14]. It was a randomized, double-blinded, placebo-controlled, primary prevention trial to determine whether daily supplementation with *dl*-α-tocopherol acetate (50 mg/day), *all-trans*-β-carotene (20 mg/day), or both, would reduce the incidence of lung or other cancers in male smokers. Men (n = 29,133) between the ages of 50 and 69 years living in southwestern Finland were recruited between the years 1985 and 1988 and assigned to 1 of 4 groups in a 2×2 factorial design. The intervention ended on April 30, 1993, however cancer ascertainment via the Finnish Cancer Registry is still ongoing.

Incident liver cancer cases (n = 208) diagnosed through December 31, 2009 were identified through the Finnish Cancer Registry using topography code 155 of the 9^th^ revision of the International Classification of Diseases (ICD). Liver disease deaths (n = 239) through December 31, 2009 were identified via the Finnish Register of Causes of Death using code 571 for ICD-8 and ICD-9 and codes K70-K77 for ICD-10. Eight participants who developed incident liver cancer but died of liver disease were only included in the liver cancer analysis. After excluding individuals with missing questionnaire data and 28 men with self-reported history of cirrhosis at baseline, 194 incident liver cancer cases and 213 liver disease deaths were available for analysis in a cohort of 27,086 persons.

Within the nested case-control study, incident liver cancer cases were diagnosed through April 30, 2002. Cases were matched 1∶1 with controls who were alive and free of cancer at the time the case was identified. The matching factors were age (within 5 years) and date of serum draw (within 30 days). After excluding missing questionnaire data and serum data, 95 cases and 103 controls were available for analysis.

### Questionnaire data

At baseline, all participants completed a general risk factor questionnaire which included queries concerning vitamin supplement use. Participants also completed a food-frequency questionnaire (FFQ) to measure usual dietary consumption within the past 12 months. The FFQ was completed by 93% of study participants. The reproducibility and validity have been previously reported [15]. Metabolite intake was estimated using food composition data from the National Public Health Institute. Total intake for each one-carbon metabolite was calculated as energy-adjusted dietary intake using the residual method plus supplemental intake [16].

### Serum one-carbon metabolite measurements

Study participants provided fasting blood samples prior to intervention-arm randomization. The serum samples were stored at −70°C prior to analysis in 2005–2006. Serum levels of folate and vitamin B12 were determined by radioimmunoassay (Bio-Rad Laboratories, Richmond, CA, USA) in the laboratory of Dr. Jacob Selhub at Tufts University, Boston, MA. The principal active form of vitamin B6, pyridoxal 5′-phosphate, was determined using the tyrosine decarboxylase apoenzyme method [17] while cysteine and homocysteine concentrations were measured using high-performance liquid chromatography (HPLC) [18]. Riboflavin was measured using HPLC with fluorimetric detection [19]. Each batch contained matched case-control pairs which placed beside one another in random order. Six blinded quality control samples which were derived from a pool of serum were also included in each batch. Within-batch coefficients of variation, based on 150 QC samples, were calculated using nested components of variance analysis on logarithmically transformed quality control data [20]. The coefficients of variation were 6.2% for folate, 4.5% for vitamin B6, 7.1% for vitamin B12, 6.3% for cysteine, 4.8% for riboflavin, and 6.2% for homocysteine.

Hepatitis B virus (HBV) status was determined by testing for the presence of hepatitis B surface antigen (HBsAg) using an enzyme immunoassay (Bio-Rad Laboratories, Redmond, WA). Hepatitis C virus (HCV) status was determined by testing for antibody to HCV (anti-HCV) using an enzyme-linked immunosorbent assay (Ortho-Clinical Diagnostics, Raritan, NJ).

### Statistical analyses

Categorization of folate, cysteine, vitamin B6, riboflavin, vitamin B12, methionine, and alcohol was based on the total adjusted intake at baseline divided into tertiles within the entire cohort. Cox proportional hazards regression models were used to calculate hazard ratios (HRs) and 95% Confidence Intervals (CIs). Models were adjusted for age (continuous age at randomization), education (<12 years, ≥12 years), smoking (<30 pack-years, 30–43 pack-years, >43 pack-years), body mass index (BMI) (<25 kg/m^2^, 25–29 kg/m^2^, ≥30 kg/m^2^), diabetes (yes, no) and study arm (α-tocopherol, β-carotene, both, neither). In addition, HRs were calculated for the relationship between alcohol consumption and liver cancer and liver disease, stratified by tertiles of one-carbon metabolite intake. Likelihood ratio tests for interaction across tertiles of one-carbon metabolite consumption were computed based on cross-product terms with alcohol use. Tests for trend were calculated by assigning each participant the median value within each tertile and entering that value as a continuous variable in the adjusted multivariate model.

In the nested case-control analysis, serum levels of folate, cysteine, vitamin B6, riboflavin, vitamin B12, homocysteine and alcohol consumption were divided into tertiles and median levels, based on the controls. Both conditional and unconditional logistic regression models were used to determine odds ratios (ORs) and 95% CIs for the associations between alcohol and one-carbon metabolites and liver cancer incidence. The models were adjusted for study arm, age, HBV and HCV. In addition, ORs were calculated for median alcohol intake and serum one-carbon metabolite level in an interaction analysis adjusting for study arm, HBV and HCV. Pearson correlation coefficients between the serum levels and intake levels of the one-carbon metabolites were also calculated.

The proportional hazard assumption was verified using a time interaction model. Statistical significance was set at P<0.05 based on two-sided tests. All statistical analyses were performed using SAS version 9.2 (SAS Institute Inc, Cary, North Carolina).

## Results

The population characteristics of the study participants by tertile of each one-carbon metabolite and alcohol consumption are shown in **Table 1**. Persons in the highest tertile of alcohol consumption had greater BMI, more years of education and had accumulated more pack-years of smoking. Persons in the highest tertiles of one-carbon metabolite intake had greater BMI and were more likely to have diabetes.

**Table 1:** Distribution of select baseline characteristics across total one-carbon metabolite intake tertiles and alcohol consumption in the ATBC Study.

	N	BMI, kg/m2 (%)			Education, years (%)		Diabetes (%)		Pack-Years (%)		
		<25	25–29	≥30	<12	≥12	No	Yes	<30	30–43	>43
**Alcohol Intake (grams/day)**											
0–5.33	8922	41.34	45.02	13.56	94.23	5.77	95.45	4.55	8.20	42.57	49.23
5.33–20.44	9119	38.38	46.62	14.97	92.09	7.91	96.03	3.97	9.10	35.39	55.51
>20.44	9045	35.77	47.29	16.88	90.58	9.42	95.91	4.09	10.55	24.51	64.94
**Folate (µg)**											
<304.87	9017	41.74	45.19	13.04	95.30	4.70	97.38	2.62	10.17	28.30	61.53
304.87 – 355.97	9023	38.22	46.70	15.04	93.62	6.38	96.08	3.92	9.12	34.98	55.90
>355.97	9046	35.49	47.05	17.34	87.97	12.03	93.94	6.06	8.58	39.07	52.35
**Cysteine (mg)**											
<1271.03	9018	44.17	44.12	11.69	91.46	8.54	98.11	1.89	9.15	30.37	60.48
1271.03 – 1419.40	9021	38.83	46.91	14.22	92.32	7.68	96.93	3.07	8.26	34.91	56.83
>1419.40	9047	32.46	47.91	19.51	93.09	6.91	92.36	7.64	10.46	37.07	52.47
**Vitamin B6 (mg)**											
<2.31	9019	41.51	45.28	13.14	94.68	5.32	97.53	2.47	10.00	32.93	57.07
2.31 – 2.74	9021	37.34	46.91	15.72	93.43	6.57	96.06	3.94	9.37	34.59	56.05
>2.74	9046	36.60	46.75	16.57	88.78	11.22	93.81	6.19	8.50	34.84	56.65
**Riboflavin (mg)**											
<2.64	9020	41.22	46.72	12.02	91.61	8.39	97.52	2.48	8.22	34.09	57.69
2.64 – 3.19	9019	39.02	46.46	14.47	94.51	5.49	96.46	3.54	9.38	34.43	56.19
>3.19	9047	35.22	45.77	18.93	90.76	9.24	93.42	6.58	10.27	33.85	55.89
**Vitamin B12 (µg)**											
<8.83	9020	40.95	45.73	13.27	94.80	5.20	96.98	3.02	9.33	36.23	54.43
8.83 –12.09	9020	37.71	46.82	15.41	92.99	7.01	95.67	4.33	9.16	34.04	56.81
>12.09	9046	36.79	46.40	16.75	89.09	10.91	94.75	5.25	9.37	32.10	58.52
**Methionine (mg)**											
<1841.83	9020	44.71	44.04	11.22	92.88	7.12	98.12	1.88	9.61	34.81	55.58
1841.83 –2106.93	9020	38.49	46.98	14.46	92.87	7.13	96.66	3.34	8.69	34.87	56.44
>2106.93	9046	32.26	47.92	19.74	91.12	8.88	92.63	7.37	9.56	32.69	57.75

**Table 2** shows the results of the analysis examining the effects of alcohol and one-carbon metabolite intake on liver cancer incidence and liver disease mortality. Increasing alcohol intake was associated with increased risk of liver cancer (p-trend = 0.02) with a significant increased risk in the highest tertile (HR 1.52, 95%CI 1.06–2.18). None of the one-carbon metabolites, however, were significantly associated with liver cancer incidence.

**Table 2:** Association between alcohol consumption and one-carbon metabolite intake and the risk of liver cancer incidence and liver disease mortality in the ATBC Study.

	Liver Cancer incidence						Liver Disease mortality					
	Cases	Non-Cases	Person-Years	HR	(95% CI)*		Cases	Non-Cases	Person-Years	HR	(95% CI)*	
**Alcohol Intake (grams/day)**												
<5.33	53	8869	143,397	1.00	Reference		20	8902	143,424	1.00	Reference	
5.33–20.44	64	9055	150,334	1.20	0.83	1.73	45	9074	150,389	2.06	1.22	3.50
>20.44	77	8968	145,640	1.52	1.06	2.18	148	8897	145,694	6.68	4.16	10.71
*P_trend_*						*0.02*						*<.0001*
**Folate (µg)**												
<304.87	53	8964	140,740	1.00	Reference		63	8954	140,770	1.00	Reference	
304.87 – 355.97	67	8956	148,253	1.21	0.84	1.74	55	8968	148,322	0.84	0.58	1.20
>355.97	74	8972	150,378	1.27	0.89	1.83	95	8951	150,414	1.42	1.02	1.96
*P_trend_*						*0.21*						*0.02*
**Cysteine (mg)**												
<1271.03	73	8945	145,643	1.00	Reference		87	8931	145,692	1.00	Reference	
1271.03 – 1419.40	56	8965	147,391	0.74	0.52	1.04	61	8960	147,429	0.70	0.50	0.97
>1419.40	65	8982	146,337	0.76	0.54	1.07	65	8982	146,385	0.74	0.54	1.03
*P_trend_*						*0.12*						*0.07*
**Vitamin B6 (mg)**												
<2.31	70	8949	141,952	1.00	Reference		63	8956	141,997	1.00	Reference	
2.31 – 2.74	48	8973	147,539	0.66	0.46	0.95	65	8956	147,570	0.96	0.68	1.36
>2.74	76	8970	149,879	0.97	0.70	1.35	85	8961	149,940	1.21	0.87	1.68
*P_trend_*						*0.81*						*0.20*
**Riboflavin (mg)**												
<2.64	64	8956	150,318	1.00	Reference		60	8960	150,355	1.00	Reference	
2.64 – 3.19	61	8958	146,600	0.96	0.68	1.36	61	8958	146,643	1.06	0.74	1.51
>3.19	69	8978	142,454	1.03	0.73	1.45	92	8955	142,508	1.60	1.15	2.22
*P_trend_*						*0.86*						*0.003*
**Vitamin B12 (µg)**												
<8.83	67	8953	147,202	1.00	Reference		56	8964	147,239	1.00	Reference	
8.83 –12.09	58	8962	145,873	0.85	0.60	1.22	60	8960	145,890	1.06	0.73	1.52
>12.09	69	8977	146,296	0.96	0.69	1.35	97	8949	146,378	1.65	1.19	2.30
*P_trend_*						*0.92*						*0.001*
**Methionine (mg)**												
<1841.83	65	8955	146,361	1.00	Reference		75	8945	146,398	1.00	Reference	
1841.83 –2106.93	59	8961	146,808	0.88	0.62	1.25	66	8954	146,833	0.86	0.62	1.19
>2106.93	70	8976	146,202	0.95	0.67	1.34	72	8974	146,275	0.90	0.65	1.24
*P_trend_*						*0.78*						*0.53*

* adjusted for age, education,smoking, BMI, diabetes and study arm

Increasing levels of alcohol intake was also significantly associated with increased risk of liver disease mortality (p-trend<0.0001). In addition, the highest tertiles of folate (HR 1.42, 95%CI 1.02–1.96), riboflavin (HR 1.60, 95%CI 1.15–2.22) and vitamin B12 (HR 1.65, 95%CI 1.19–2.30) intake were all associated with an increased risk of liver disease mortality. In contrast, cysteine intake was inversely, but not significantly, associated with risk (p-trend = 0.07). Intakes of vitamin B6 and methionine had no association with liver disease mortality.

In addition, interactions between alcohol and one-carbon metabolites were examined for effects on liver cancer incidence and liver disease mortality. None of the interactions were statistically significant (data not shown).

In the nested case-control analysis, the relationships of alcohol consumption and serum one-carbon metabolite levels with liver cancer were examined. As shown in **Table 3**, the results of the unconditional logistic regression analysis found none of the relationships were significant. The conditional logistic regression analysis also found no significant relationships (data not shown). In addition, the interactions between alcohol and serum one- carbon metabolites were examined in relationship to liver cancer, but none were statistically significant (data not shown).

**Table 3:** Association between alcohol consumption and one-carbon metabolite serum levels and the risk of liver cancer incidence in the ATBC Study.

	Cases	Controls	OR	(95% CI)*	
**Alcohol Intake (grams/day)**					
<4.15	26	34	1.00	Reference	
4.15–13.80	24	35	0.92	0.40	2.14
>13.80	45	34	1.24	0.54	2.85
*P_trend_*					*0.54*
**Folate (nmol/L)**					
<3.08	33	34	1.00	Reference	
3.08 – 4.42	34	36	1.32	0.61	2.87
>4.42	28	33	0.82	0.43	1.92
*P_trend_*					*0.65*
**Cysteine (nmol/mL)**					
<280.50	29	34	1.00	Reference	
280.50 – 330.48	35	35	1.17	0.52	2.61
>330.48	31	34	1.28	0.59	2.88
*P_trend_*					*0.55*
**Vitamin B6 (pmol/mL)**					
<18.58	43	34	1.00	Reference	
18.58 – 30.27	24	35	0.53	0.24	1.21
>30.27	28	34	0.68	0.31	1.50
*P_trend_*					*0.48*
**Riboflavin (pmol/mL)**					
<7.16	34	34	1.00	Reference	
7.16 – 9.86	26	35	0.63	0.28	1.44
>9.86	35	34	0.86	0.39	1.90
*P_trend_*					*0.81*
**Vitamin B12 (pmol/L)**					
<423.14	31	34	1.00	Reference	
423.14 –562.67	34	35	1.31	0.58	2.96
>562.67	30	34	0.74	0.33	1.62
*P_trend_*					*0.46*
**Homocysteine (nmol/mL)**					
<12.24	32	34	1.00	Reference	
12.24–14.74	23	37	0.80	0.35	1.84
>14.74	40	32	1.52	0.69	3.36
*P_trend_*					*0.22*

* adjusted for age,education, smoking, BMI, diabetes, study arm, HBV, HCV

The Pearson correlation coefficients examining the correlation between intake and serum levels one-carbon metabolite levels were all statistically significant except for cysteine (correlation coefficient 0.10, p = 0.18), with coefficients ranging from 0.31 for vitamin B6 to 0.64 for vitamin B12.

## Discussion

In the current study, alcohol intake was positively associated with an increased risk of both liver cancer incidence and liver disease mortality, as anticipated. None of the one-carbon metabolites were significantly associated with liver cancer risk, however. Conversely, folate, riboflavin and vitamin B12 intakes were associated with an increased risk of liver disease mortality. The examination of serum levels of one-carbon metabolites in relation to liver cancer found no significant relationships.

One-carbon metabolism, conducted by linked biochemical pathways that involve the interactions of B vitamins, homocysteine and methionine, is critical for the synthesis of both DNA and S-adenosylmethionine, a methyl donor for many reactions including DNA methylation [21]. While folate and methionine are important dietary sources of methyl groups, alcohol is known folate antagonist that can interfere with both folate absorption and metabolism [22], [23].

While several experimental animal studies have shown that depleted one-carbon metabolites are associated with hepatocellular carcinoma (HCC) risk, only one human case-control study to date has found serum folate levels to be inversely associated with liver cancer [24]. In addition, an interaction between alcohol consumption and folate intake on risk of HCC has previously been found by our group [13]. The prior study, conducted in the NIH-AARP Diet and Health Study cohort in the United States, suggested that higher folate intake could ameliorate the effect of alcohol consumption on the development of HCC. Those results were not directly replicated in the present study, perhaps due to differences in study populations and overall folate intake. In the NIH-AARP study conducted in the U.S., the tertiles of folate intake were 1) <419.2 µg/day, 2) 419.2–736.9 µg/day, 3) >736.9 µg/day while in the present study, the tertiles of folate intake were 1) <304.9 µg/day, 2) 304.9–356.0 µg/day, 3) >356.0 µg/day. The notably lower folate intake levels in the ATBC study conducted in Finland may be due to lower folate intake in Finland than in the U.S. In addition, Finland does not practice dietary folate fortification. In contrast, the implementation of the U.S. folate fortification program in 1998 has resulted in increased mean folic acid intakes in the United States of approximately 190 µg/day [25].

In addition to differences in dietary patterns, smoking and drinking patterns also differed in the NIH-AARP and ATBC studies. All men in the ATBC study smoked 5 or more cigarettes per day at baseline, while in the NIH-AARP study 35% of participants were never smokers. Smokers are known to have lower serum levels of folate, vitamin B6, and vitamin B12, possibly due to a negative effect of components in tobacco smoke on one-carbon metabolism [26]–[28]. In the ATBC study, 11% of participants were non-drinkers and 11% reported consuming more than three drinks of alcohol per day (42 grams), while in the NIH-AARP cohort 24% of participants reported no alcohol consumption and only 8% reported drinking more than 3 drinks per day. The overall increase in percentage of drinkers and drinkers with greater alcohol consumption may have further decreased levels of one-carbon metabolites.

While intakes of one-carbon metabolites were not associated with the development of liver cancer, intakes of several metabolites were associated with increased risk of liver disease mortality. It is possible that these findings are due to men with existing liver disease at baseline being counseled to increase their intakes of B vitamins to prevent deficiencies associated with liver damage. Although men with cirrhosis were excluded from study participation, other forms of liver disease were not exclusionary criteria.

Strengths of this study include using both a prospective design to examine one-carbon metabolite intake, and a nested case-control design to examine serum levels of one-carbon metabolites. The present study was also able to adjust for HBV and HCV which are known risk factors for liver disease and liver cancer. Study limitations include having a rather small case number, a population restricted to male cigarette smokers and the determination of dietary intake by a food-frequency questionnaire rather than a 24 hour dietary record. In addition, the inclusion of ex-drinkers in the non-drinking category may have biased the estimates toward the null. As a result, caution should be exercised when attempting to extrapolate the results to other populations.

As a prior study in the NIH-AARP cohort found an inverse association between folate intake and risk of hepatocellular carcinoma among alcohol consumers, but the current study did not, further research on the topic is warranted.

## References

[1] Schutze M, Boeing H, Pischon T, Rehm J, Kehoe T, et al (2011) Alcohol attributable burden of incidence of cancer in eight European countries based on results from prospective cohort study. BMJ 342: d1584. 21474525 10.1136/bmj.d1584 PMC3072472

[2] Boffetta P, Hashibe M, La Vecchia C, Zatonski W, Rehm J (2006) The burden of cancer attributable to alcohol drinking. Int J Cancer 119: 884–887. 16557583 10.1002/ijc.21903

[3] Dong C, Yoon YH, Chen CM, Yi HY (2011) Heavy alcohol use and premature death from hepatocellular carcinoma in the United States, 1999-2006. J Stud Alcohol Drugs 72: 892–902. 22051203 10.15288/jsad.2011.72.892 PMC3211960

[4] Shimazu T, Sasazuki S, Wakai K, Tamakoshi A, Tsuji I, et al (2012) Alcohol drinking and primary liver cancer: a pooled analysis of four Japanese cohort studies. Int J Cancer 130: 2645–2653. 21702041 10.1002/ijc.26255

[5] Trichopoulos D, Bamia C, Lagiou P, Fedirko V, Trepo E, et al (2011) Hepatocellular carcinoma risk factors and disease burden in a European cohort: a nested case-control study. J Natl Cancer Inst 103: 1686–1695. 22021666 10.1093/jnci/djr395 PMC3216968

[6] Choi SW, Mason JB (2000) Folate and carcinogenesis: an integrated scheme. J Nutr 130: 129–132. 10720158 10.1093/jn/130.2.129

[7] Kim YI (1999) Folate and carcinogenesis: evidence, mechanisms, and implications. J Nutr Biochem 10: 66–88. 15539274 10.1016/s0955-2863(98)00074-6

[8] Stover PJ (2004) Physiology of folate and vitamin B12 in health and disease. Nutr Rev 62: S3–12; discussion S13 15298442 10.1111/j.1753-4887.2004.tb00070.x

[9] Friso S, Choi SW (2005) Gene-nutrient interactions in one-carbon metabolism. Curr Drug Metab 6: 37–46. 15720206 10.2174/1389200052997339

[10] Heinen MM, Verhage BA, Ambergen TA, Goldbohm RA, van den Brandt PA (2009) Alcohol consumption and risk of pancreatic cancer in the Netherlands cohort study. Am J Epidemiol 169: 1233–1242. 19318612 10.1093/aje/kwp028

[11] Stolzenberg-Solomon RZ, Chang SC, Leitzmann MF, Johnson KA, Johnson C, et al (2006) Folate intake, alcohol use, and postmenopausal breast cancer risk in the Prostate, Lung, Colorectal, and Ovarian Cancer Screening Trial. Am J Clin Nutr 83: 895–904. 16600944 10.1093/ajcn/83.4.895

[12] Ibiebele TI, Hughes MC, Pandeya N, Zhao Z, Montgomery G, et al (2011) High intake of folate from food sources is associated with reduced risk of esophageal cancer in an Australian population. J Nutr 141: 274–283. 21178085 10.3945/jn.110.131235 PMC3021447

[13] Persson EC, Schwartz LM, Park Y, Trabert B, Hollenbeck AR, et al (2013) Alcohol consumption, folate intake, hepatocellular carcinoma, and liver disease mortality. Cancer Epidemiol Biomarkers Prev 22: 415–421. 23307533 10.1158/1055-9965.EPI-12-1169 PMC3596467

[14] The ATBC Cancer Prevention Study Group (1994) The alpha-tocopherol, beta-carotene lung cancer prevention study: design, methods, participant characteristics, and compliance. The ATBC Cancer Prevention Study Group. Ann Epidemiol 4: 1–10. 8205268 10.1016/1047-2797(94)90036-1

[15] Pietinen P, Hartman AM, Haapa E, Rasanen L, Haapakoski J, et al (1988) Reproducibility and validity of dietary assessment instruments. I. A self-administered food use questionnaire with a portion size picture booklet. Am J Epidemiol 128: 655–666. 2458036 10.1093/oxfordjournals.aje.a115013

[16] Willett W, Stampfer MJ (1986) Total energy intake: implications for epidemiologic analyses. Am J Epidemiol 124: 17–27. 3521261 10.1093/oxfordjournals.aje.a114366

[17] Shin-Buehring YS, Stuempfig L, Pouget E, Rahm P, Schaub J (1981) Characterization of galactose-1-phosphate uridyl-transferase and galactokinase in human organs from the fetus and adult. Clin Chim Acta 112: 257–265. 6263522 10.1016/0009-8981(81)90448-4

[18] Araki A, Sako Y (1987) Determination of free and total homocysteine in human plasma by high-performance liquid chromatography with fluorescence detection. J Chromatogr 422: 43–52. 3437026 10.1016/0378-4347(87)80438-3

[19] Zempleni J, Link G, Kubler W (1992) The transport of thiamine, riboflavin and pyridoxal 5′-phosphate by human placenta. Int J Vitam Nutr Res 62: 165–172. 1517040

[20] Fears TR, Ziegler RG, Donaldson JL, Falk RT, Hoover RN, et al (2002) Reproducibility studies and interlaboratory concordance for androgen assays of male plasma hormone levels. Cancer Epidemiol Biomarkers Prev 11: 785–789. 12163335

[21] Anderson OS, Sant KE, Dolinoy DC (2012) Nutrition and epigenetics: an interplay of dietary methyl donors, one-carbon metabolism and DNA methylation. J Nutr Biochem 23: 853–859. 22749138 10.1016/j.jnutbio.2012.03.003 PMC3405985

[22] Halsted CH, Villanueva JA, Devlin AM, Chandler CJ (2002) Metabolic interactions of alcohol and folate. J Nutr 132: 2367S–2372S. 12163694 10.1093/jn/132.8.2367S

[23] Seitz HK, Stickel F (2007) Molecular mechanisms of alcohol-mediated carcinogenesis. Nat Rev Cancer 7: 599–612. 17646865 10.1038/nrc2191

[24] Welzel TM, Katki HA, Sakoda LC, Evans AA, London WT, et al (2007) Blood folate levels and risk of liver damage and hepatocellular carcinoma in a prospective high-risk cohort. Cancer Epidemiol Biomarkers Prev 16: 1279–1282. 17548697 10.1158/1055-9965.EPI-06-0853

[25] Choumenkovitch SF, Selhub J, Wilson PW, Rader JI, Rosenberg IH, et al (2002) Folic acid intake from fortification in United States exceeds predictions. J Nutr 132: 2792–2798. 12221247 10.1093/jn/132.9.2792

[26] Piyathilake CJ, Macaluso M, Hine RJ, Richards EW, Krumdieck CL (1994) Local and systemic effects of cigarette smoking on folate and vitamin B-12. Am J Clin Nutr 60: 559–566. 8092091 10.1093/ajcn/60.4.559

[27] Gabriel HE, Crott JW, Ghandour H, Dallal GE, Choi SW, et al (2006) Chronic cigarette smoking is associated with diminished folate status, altered folate form distribution, and increased genetic damage in the buccal mucosa of healthy adults. Am J Clin Nutr 83: 835–841. 16600936 10.1093/ajcn/83.4.835

[28] Mannino DM, Mulinare J, Ford ES, Schwartz J (2003) Tobacco smoke exposure and decreased serum and red blood cell folate levels: data from the Third National Health and Nutrition Examination Survey. Nicotine Tob Res 5: 357–362. 12791531 10.1080/1462220031000094330

